# Reproducibility and Validity of a Semi-Quantitative Food Frequency Questionnaire for Assessing Dietary Intake of Vegetarians and Omnivores in Harbin, China

**DOI:** 10.3390/nu14193975

**Published:** 2022-09-24

**Authors:** Changbao Sun, Qingyun Wang, Cong Xu, Wan Wang, Jiage Ma, Liya Gu, Zhijing Liu, Juncai Hou, Zhanmei Jiang

**Affiliations:** 1 College of Food Science, Northeast Agricultural University, Harbin 150030, China; 2 College of Food and Biological Engineering, Qiqihar University, Qiqihar 161006, China; 3 Beidahuang Wandashan Dairy Co., Ltd., Harbin 150090, China

**Keywords:** semi-quantitative food frequency questionnaire, vegetarians, omnivores, reproducibility, validity

## Abstract

This study aims to evaluate the reproducibility and validity of a semi-quantitative food frequency questionnaire (SQFFQ) developed for vegetarians and omnivores in Harbin, China. Participants (36 vegetarians and 64 omnivores) administered SQFFQ at baseline (SQFFQ1) and six months later (SQFFQ2) to assess the reproducibility. The 24 h recalls (24 HRs) for three consecutive days were completed between the administrations of two SQFFQs to determine the validity. For reproducibility, Pearson correlation coefficients between SQFFQ1 and SQFFQ2 for vegetarians and omnivores were 0.45~0.88 and 0.44~0.84, respectively. For validity, unadjusted Pearson correlation coefficients were 0.46~0.83 with an average of 0.63 and 0.43~0.86 with an average of 0.61, respectively; energy-adjusted Pearson correlation coefficients were 0.43~0.82 with an average of 0.61 and 0.40~0.85 with an average of 0.59, respectively. Majority of the correlation coefficients for food groups and macronutrients decreased or remained unchanged after energy adjustment. Furthermore, all correlations were statistically significant (*p* < 0.05). Bland–Altman plots also showed reasonably acceptable agreement between the two methods. In conclusion, the SQFFQ developed in this study has reasonably acceptable reproducibility and validity.

## 1. Introduction

As global public health issues, chronic diseases, especially obesity, diabetes, cancer and cardiovascular diseases, have attracted more and more attention. Epidemiological studies have suggested that dietary habits are likely to be related to the occurrence of chronic disease. A vegetarian diet can reduce the incidence rate of obesity [1], diabetes [2], cancer [3] and cardiovascular diseases [4], while an omnivorous diet may have the opposite effect [5]. In order to determine the relationship between dietary habits and chronic diseases, it is necessary to clearly understand and reliably assess the dietary intake of vegetarians and omnivores to improve their health status through effective dietary interventions. Weighed food records are a method that can accurately measure dietary intake, but the main limitation of this method is that it is time-consuming and more suitable for short-term individual dietary intake surveys [6]. The semi-quantitative food frequency questionnaire (SQFFQ) is a widely used method to assess dietary intake over various periods in epidemiological research because of its timesaving, low cost, simple operation and high response rate, and the data collected reflect the dietary intake in the past for a long time, which is more valuable than the short-term data [7,8]. Since an SQFFQ is prone to some degree of measurement error that may attenuate associations between dietary intake and disease. All newly developed SQFFQs need to be evaluated for reproducibility and validity [9,10,11]. Reproducibility refers to the consistency and reliability of the survey results, that is the consistency between the two same SQFFQs. Validity refers to the effectiveness and authenticity of the survey results, that is the consistency between the SQFFQ and the actual intake. Although there is no ‘gold standard’ for measuring dietary intake, other dietary assessment methods can offer valuable insights [12]. The 24 HRs for three consecutive days are often used as a reference dietary assessment method to evaluate the validity of the SQFFQ [13,14,15].

A vegetarian diet is characterized by its high dietary fiber content primarily from plant foods (i.e., vegetables, fruit, beans and coarse cereals) rather than high-fat content animal foods (i.e., meat, poultry and fish), which is clearly shown to be beneficial to human health [16,17]. In recent years, the vegetarian population has gradually increased in China [18]. Some SQFFQs for monks have been developed to estimate the dietary intake [19,20,21], but research on the dietary intake of ordinary vegetarians and omnivores in Harbin, China, has not been reported yet, where different dietary preferences and dietary habits existed from other areas. The SQFFQs should be tailored to the target population because of the vast difference in food items and food intake depending on ethnic, social and cultural backgrounds [22,23]. Therefore, we newly developed an SQFFQ to estimate the dietary intake of vegetarians and omnivores in Harbin, China.

Therefore, the present study aimed to estimate the reproducibility and validity of the newly developed SQFFQ for evaluating the dietary intake of vegetarians and omnivores and provide the theoretical basis for determining the relationship between dietary habits and chronic diseases.

## 2. Methods

### 2.1. Ethics Statement

This study was approved by the Research Ethics Committee of Northeast Agricultural University (Harbin, China). Written informed consent was obtained from all the participants before participating in the dietary survey.

### 2.2. Study Participants

The participants in the present study were recruited through advertisements, email and telephone in the area of Harbin, China. For inclusion in the study, participants were required to be 25~40 years of age, without chronic, nutritional, or infectious diseases; not pregnant nor breastfeeding; with no smoking or drinking habits; and vegetarians who have been on a vegetarian diet for at least one year [24,25]. Written informed consent was obtained from all participants for participation in this study. We collected information about participants’ age, education level and employment status. Height was measured without shoes at 0.1 cm using a research-grade digital stadiometer (Model: HT-DM40, Faenza, Italy). Weight was measured in light clothing without shoes to the nearest 0.1 kg with a portable digital scale (Model: Yolanda-CS10A, Shenzhen, China). Body mass index (BMI) was calculated according to the following formula [26]: BMI = weight (kg)/height (m^2^). In the end, one hundred and twenty-one participants (46 vegetarians and 75 omnivores) were recruited to participate in this study.

### 2.3. Study Design

**Figure 1:**
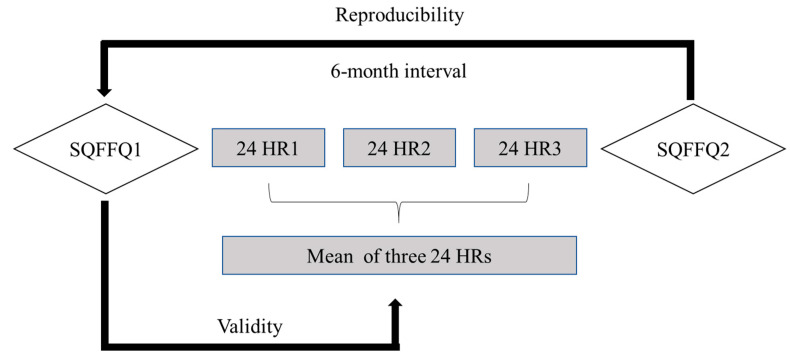
Study design and schedule used in this study. A 22 food groups semi-quantitative food frequency questionnaire (SQFFQ) was administrated at the baseline (SQFFQ1) and 6 months later (SQFFQ2) to vegetarians and omnivores by trained interviewers with a face-to-face approach. The 24 h dietary recalls (24 HRs) for three consecutive days (including two weekdays and one weekend day) were performed by participants between SQFFQ1 and SQFFQ2 to recall the items and portion sizes of all foods that they consumed from the last day (22:00) to the next day (22:00). The reproducibility was tested by comparing the results from two SQFFQs, and the validity was assessed by comparing the data obtained from the SQFFQ1 and the mean 24 HRs.

The study started in July 2017 and lasted for the subsequent six months. During the study period, participants were required to complete two administrations of SQFFQs and one 24 HRs for three consecutive days including two weekdays and one weekend day [21]. In the reproducibility study, the SQFFQ1 was administrated by a trained interviewer and the SQFFQ2 at the following visit six-month later [27]. To validate the SQFFQ, the 24 HRs for three consecutive days were completed between the intervals of two SQFFQs [28]. In return, each participant will receive a detailed dietary assessment and personalized dietary guidance based on the results of the nutrient intake analysis of the participant. The study design and schedule used are shown in Figure 1.

### 2.4. Semi-Quantitative Food Frequency Questionnaire

The SQFFQ was developed based on the methodology proposed by Willett [6]. The SQFFQ consisted of three parts, including the food items list, the frequency of food consumption, and the amount of food consumed each time. There were 116 items on the food list, which were divided into 22 groups (Table 1). Each food item was based on the dietary guidelines for Chinese residents. [26], the National Health and Dietary Survey in China [29] and the dietary habits of the Chinese local vegetarians and omnivores. The frequency options provided in the SQFFQ were (1) none/no consumption; (2) Number of times per day; (3) Number of times per week; (4) Number of times per month; (5) Number of times per year. The average amount consumed each time was filled in “gram, g” or “milliliter, mL”. To improve the accuracy of participants’ estimation of food weight, we provided plastic food models and photos of standard food portion size to facilitate the assessment of food weight [30]. For seasonal foods (e.g., watermelon, grape, and cucumber), participants were asked to recall how often they ate these foods during the season, and then interviewers converted consumption frequency during the season to an average consumption frequency over a year [31]. For example, the participants ate watermelon for 3 months (June to August) in the past year, consuming 1000 g each time and 3 times a week on average, record “1000 g” in the column of average consumption per time and “36” in the column of “annual” eating times. The mean intake of each food item per day was calculated by multiplying the frequency of daily food consumption and the amount of food consumed each time in g/d or mL/d [32].

### 2.5. 24 Hour Dietary Recall

Each participant was asked to complete one 24 HRs for three consecutive days during the intervals of two SQFFQs. The three 24 HRs included two weekdays and one weekend day. Participants were required to recall the items and portion sizes of all foods consumed during the past 24 h from the last day (22:00) to the next day (22:00). The mixed dishes were converted into single food items. The recalled food items were assigned to the corresponding food groups as defined by the SQFFQ. Trained interviewers administered the SQFFQs and 24 HRs through face-to-face interviews. The mean 24 HRs were used as a reference method to validate the SQFFQ [28]. During the whole study period, each participant corresponded to the same interviewer to reduce possible bias.

### 2.6. Data Cleaning

Participants who did not complete the SQFFQs or 24 HRs were excluded from the analyses. Participants with implausible energy intakes (< 600 kcal/day or > 4000 kcal/day) were also excluded [19,33]. The total energy intake of each participant was calculated based on the Chinese food composition tables [34].

### 2.7. Statistical Analysis

All statistical analyses were performed with SPSS 20.0. A value of *p* < 0.05 was considered to be statistically significant. Categorical variable data, such as gender, education level and employment status were represented by frequency (*n*) and percentage (%). Continuous variable data, such as age, height, weight and BMI, were represented by the mean and standard deviation (SD). The daily intake of each food item was determined based on the average consumption frequency and the amount of each food item [35]. Macronutrient intake for each food item was calculated as the daily intake of each food item multiplied by nutrient per 100 g [36]. The macronutrient composition of foods can be found in the Chinese Food Composition Tables [34]. Descriptive statistics for energy, macronutrients and food intake are presented as mean and standard deviation (SD), median and interquartile ranges, respectively. Differences in food and macronutrient intake between two SQFFQs, and between the SQFFQ1 and the mean 24 HRs, were compared using the Wilcoxon signed rank test [20]. Pearson correlation coefficients were calculated to access the association between average daily intake of nutrients and food [37]. The correlation coefficients of 0.10~0.39, 0.40~0.69, 0.70~0.89 and 0.90~1.00 represent a weak, moderate, strong and very strong correlation, respectively [38]. Energy-adjusted intakes of food and macronutrients were calculated by using the residual method [39] to remove the person variation caused by day-to-day fluctuations and seasonal variations and were used to calculate correlation coefficients for assessing the relationship between the SQFFQ1 and the mean 24 HRs. For visualization, Bland–Altman plots were drawn to examine the agreement between the SQFFQ1 and the mean 24 HRs for energy and macronutrients [40]. A good agreement was defined as having no more than 10% of the points exceeding the 95% limits of agreement and being close to the mean line [32].

## 3. Results

All enrolled participants (*n* = 121) completed the questionnaires, in which participants who did not satisfactorily complete the SQFFQs or 24 HRs (7 vegetarians and 5 omnivores) and had implausible energy intake (3 vegetarians and 6 omnivores) were excluded from the analyses, and 100 (82.6%) subjects completed the study.

The characteristics of the 100 subjects are shown in Table 2. The mean age was 32.8 ± 4.8 years, ranging from 25 to 45 years and 52.0% were women. The mean height was 167.0 ± 7.4 cm. The mean weight was 65.1 ± 5.9 kg, and the mean BMI was 23.1 ± 3.1 kg/m^2^, ranging from 18.8 to 30.0 kg/m^2^. In total, 18.0% of the subjects had a university degree or above; 72% of the subjects had employment status. There was no significant difference in age, height, weight and BMI between vegetarians and omnivores (*p* > 0.05).

### 3.1. Reproducibility

As shown in Table 3, in the comparison of the intake of foods, energy and macronutrients from two SQFFQs, for vegetarians, the intake of rice, flour food, buns, eggs, dark vegetables, fruits, nuts, beverages, energy, protein, fat and carbohydrates was higher when estimated by SQFFQ2 than by SQFFQ1; for omnivores, the intake of buns, pastry food, fried food, red meat, processed meat, freshwater fish, seafood, bean products, light vegetables, mushrooms, beverages, energy, protein, fat and carbohydrates was higher when estimated by SQFFQ2 than by SQFFQ1. The differences between SQFFQ1 and SQFFQ2 for vegetarians and omnivores were 0~9.7% and 0.1%~15.8%, respectively. Through the Wilcoxon rank-sum test, there was no significant difference in foods energy and macronutrient intake between SQFFQ1 and SQFFQ2 (*p* > 0.05). The Pearson correlation coefficients of the two SQFFQs on vegetarians ranged from 0.45 for eggs to 0.88 for fruits with an average of 0.65; the Pearson correlation coefficients of the two SQFFQs on omnivores ranged from 0.44 for coarse cereals to 0.84 for dairy with an average of 0.64. Furthermore, all correlations were statistically significant (*p* < 0.05). It shows that the survey results of SQFFQ1 and SQFFQ2 are consistently indicating reasonably acceptable reproducibility.

### 3.2. Validity

Table 4 shows the validity of food, energy and macronutrient intake between the SQFFQ1 and the mean 24 HRs. Compared with the mean 24 HRs, except for red meat, poultry, processed meat, freshwater fish and seafood, food groups (i.e., rice, flour food, buns, eggs, bean products and dark vegetables), energy and macronutrients of vegetarians were underestimated in SQFFQ1, with a different rate of 1.7%~9.5%, and other food groups were overestimated, with a different rate of 0.3%~13.5%. Among omnivores, food groups (i.e., rice, porridge, fried food, coarse cereals, potato, dairy, eggs, poultry, freshwater fish, dark vegetables, light vegetables, mushrooms and fruits) were overestimated, with a different rate of 0.8%~13.9%, and others were underestimated, with a different rate of 0.4%~17.2%. Although there was underestimation and overestimation, no significant difference was observed for the food groups, energy and macronutrients between SQFFQ1 and the mean 24 HRs (*p* > 0.05). The unadjusted Pearson correlation coefficients of the SQFFQ1 and the mean 24 HRs on vegetarians and omnivores were 0.46~0.83 with an average of 0.63 and 0.43~0.86 with an average of 0.61, respectively. All correlations were statistically significant (*p* < 0.05). Most of correlation coefficients for food groups and macronutrients decreased or remained unchanged after energy adjustment. The energy-adjusted Pearson correlation coefficient of the SQFFQ1 and the mean 24 HRs on vegetarians and omnivores were 0.43~0.82 with an average of 0.61 and 0.40~0.85 with an average of 0.59, respectively. All correlations were statistically significant (*p* < 0.05). It shows that the survey results of SQFFQ1 and 24 HRs are consistent indicating reasonably acceptable validity.

### 3.3. Bland–Altman Analyses

**Figure 2:**
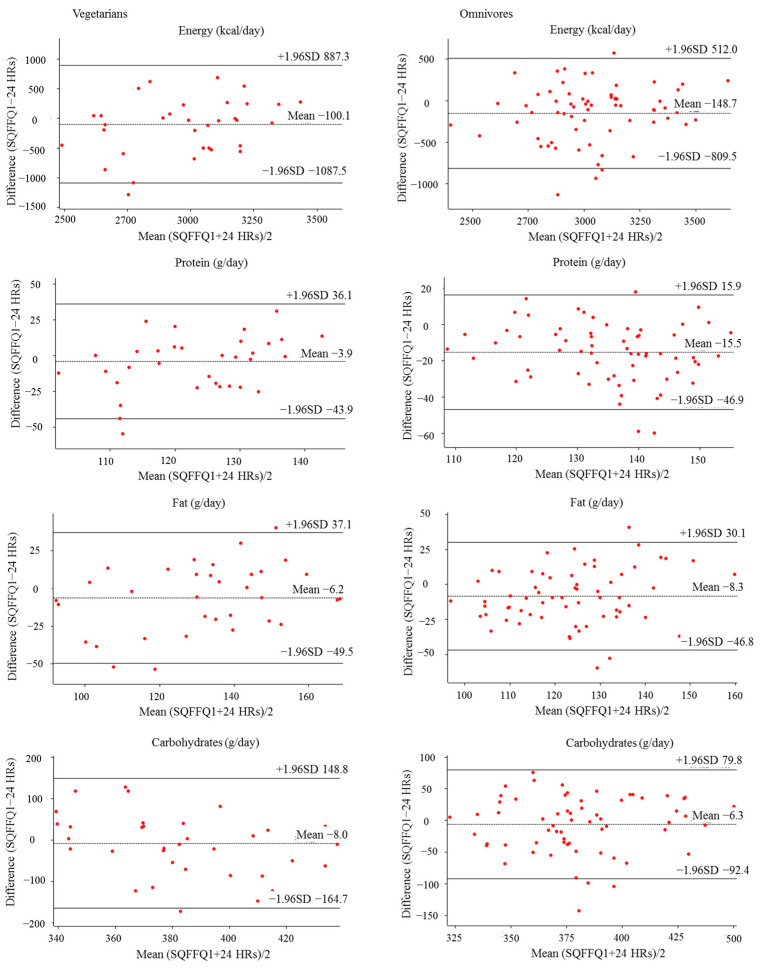
Bland–Altman plot represents the agreement between the SQFFQ1 and 24 HRs for energy and macronutrients. The dashed line represents the average difference between the two methods, while the solid line represents the distance between the mean of the difference ± 1.96 times standard deviations.

Bland–Altman plots are a graphical representation that shows the agreement between the SQFFQ1 and 24 HRs for energy and macronutrients, some of which are shown in Figure 2. The horizontal axis represents the mean total intake of energy and macronutrients from both SQFFQ1 and 24 HRs, whereas the vertical axis represents the difference in energy and macronutrient intake between the SQFFQ1 and 24 HRs. The dashed line represents the average difference between the two methods, while the solid line represents the distance between the mean of the difference ± 1.96 times standard deviations. A good agreement was defined as having no more than 10% of the points exceeding the 95% limits of agreement and being close to the mean line [32]. As shown in Figure 1, except for a few points outside the 95% limits of agreement, most of the points were within the 95% limits of agreement, and most of them were close to the mean line.

## 4. Discussion

In order to determine the relationship between dietary habits and chronic diseases, an SQFFQ consisting of 116 food items was developed to assess the dietary intake of vegetarians and omnivores in Harbin, China. The SQFFQ developed in this study was considered to have an optimal number of food items according to Cade’s suggestion that the number of food items ranges from 5 to 350 [15]. In the present study, we evaluated the reproducibility and validity of the SQFFQ. Reproducibility means that the same questionnaire is used to measure the same subject twice at different time points. The larger the correlation coefficient of the data obtained from the two surveys, the better the reproducibility of the questionnaire [12]. With regard to time frame, varying time intervals between SQFFQ1 and SQFFQ2, from 15 days to several years, have been reported in previous studies [11,41]. The time interval between the two questionnaires should be as long as the respondents cannot remember the results of the last answer and as short as the dietary habits of the respondents do not change during the two questionnaires. Some researchers believe that the interval of half a year to one year is better [42]. Therefore, the time interval between the two dietary surveys in this study is 6 months, and SQFFQ1 and SQFFQ2 are obtained, respectively. However, the time reference can reflect changes in intake caused by seasonality, which may have occurred in this study, possibly lowering true correlations, especially for fruits and vegetables.

In the reproducibility study, the results showed that there was no statistically significant difference between SQFFQ1 and SQFFQ2. Pearson correlation coefficients of the two SQFFQs on vegetarians and omnivores were 0.45~0.88 and 0.44~0.84, respectively (*p* < 0.05). It was similar to the reports of a previous study [43]. Among them, the correlation coefficients of coarse cereals (0.81), dark vegetables (0.85), light vegetables (0.84) and fruits (0.88) of vegetarians were higher than those of the others; the correlation coefficients of red meat (0.84), poultry (0.83), processed meat (0.79) and seafood (0.78) of omnivores were higher than those of the others. A possible reason for the higher correlation coefficients could be relative to their dietary habits. Some researchers believe that the correlation coefficient between dietary survey methods can reach more than 0.4, and if the correlation is meaningful, it can be considered that the survey results are consistent indicating reasonably acceptable reproducibility [44].

The validity refers to the effectiveness and authenticity of the survey results, that is, the consistency between the data obtained by SQFFQ and the actual intake data. At present, no assessment methods can accurately estimate dietary intake. Therefore, validity evaluation can only be achieved by comparing the results of the SQFFQ with a relatively accurate assessment method. The 24 HRs for three consecutive days are often used as a reference method [45,46], because of their no impact on the measurement of the SQFFQ, and the measurement error between the two methods is irrelevant [47]. In this study, the statistical and validity analysis showed that there was no statistically significant difference between SQFFQ1 and 24 HRs in the food, energy and macronutrient intake of vegetarians and omnivores. The unadjusted Pearson correlation coefficients of the two methods on vegetarians and omnivores were 0.46~0.83 and 0.43~0.86, respectively (*p* < 0.05). The adjusted Pearson correlation coefficients of the two methods on vegetarians and omnivores were 0.43~0.82 and 0.40~0.85, respectively (*p* < 0.05). The correlation coefficients of most foods and macronutrients ranged from 0.4 to 0.7, showing moderate agreement. Similar to a previous FFQ study [48], correlation coefficients of most food and nutrients in this study decreased after energy adjustment. This may be due to the large differences in energy intake among individuals. Similarly, variability was associated with an overestimation or an underestimation of systematic errors. In addition, we used the Bland–Altman plots to evaluate the validity of the SQFFQ and 24 HRs. A good agreement was defined as having no more than 10% of the points exceeding the 95% limits of agreement and being close to the mean line [32]. Bland–Altman consistency analysis showed that the SQFFQ1 and 24 HRs in vegetarians and omnivores are good consistency, indicating that the SQFFQ has reasonably acceptable validity.

## 5. Strength and Limitations

FFQs and other forms of memory-based dietary assessment methods are useful tools in epidemiological studies to understand subjects’ dietary intake [49]. Even though the limitation of these assessment methods is acknowledged, SQFFQs remain until nowadays the most used dietary assessment method to study dietary patterns.

The main strength of this study is that the trained interviewers administered the SQFFQs and 24 HRs through face-to-face interviews, and each participant corresponded to the same interviewer to minimize possible bias during the whole study period. Moreover, to improve the accuracy of participants’ estimation of food weight, we provided plastic food models and photos of standard food portion size to facilitate the assessment of food weight.

On the other hand, there were a few limitations to this study. Willett [6] has suggested a sample size of 100 to 200 as reasonable for validation studies; however, this study excluded many categories of population based on recruitment criteria and reasonable questionnaire, and these exclusions might result in a relatively small sample size for validity assessment. The 24 HRs might not be adequate to reflect the seasonal effects and other poorly defined fluctuations in dietary consumption. For seasonal foods, factors such as forgetfulness and assessment of food portion size can cause food underestimation [50]. In addition, biological markers are used as the reference methods for validity assessment. We did not use biomarkers to assess dietary intake since they are affected by bioavailability and absorption which may lead to underestimation [51], which means that we can only rely on 24 HRs to assess the validity of SQFFQs.

## 6. Conclusions

The results demonstrated that the SQFFQ has reasonably acceptable reproducibility and validity for assessing the dietary consumption of vegetarians and omnivores in Harbin, China. Based on the present study, this SQFFQ may likely be applied to epidemiological investigations of the relationship between the dietary intake of vegetarians and omnivores and chronic diseases in similar areas.

## Figures and Tables

**Table 1:** Food and drinks were used in the study.

Food Groups (22)	Food Items (116)
Rice	rice, glutinous rice, rice noodles
Porridge	rice porridge, pickled rice
Flour food	steamed bread, steamed roll, noodle
Buns	steamed stuffed bun, dumplings, pie, wonton
Pastry food	cake, chocolates, bread, biscuit
Fried food	deep-fried dough sticks, French fries, oil cake
Coarse cereals	corn, brown rice, millet, job’s tears, oats
Potato	sweet potato, potato, taro, Chinese yam
Dairy	fresh milk, powdered milk, soymilk, yogurt
Eggs	quail egg, chicken eggs, duck eggs, goose eggs
Red meat	pork, beef, mutton
Poultry	chicken meat, duck meat, goose meat
Processed meat	sausage, luncheon meat, ham, bacon
Freshwater fish	grass carp, silver carp, crucian carp, shrimp
Seafood	hairtail, pomfret, yellow croaker, shrimp
Bean products	fresh tofu, soybean milk, soybean sprout, dried tofu
Dark vegetables	spinach, eggplant, tomato, romaine lettuce, pepper, towel gourd, celery, leek, rape, pumpkin, coriander, broccoli, lettuce, garlic bolt, endive, chrysanthemum, beans, amaranth, carrot, purple cabbage
Light vegetables	cabbage, radish, cucumber, cauliflower, bean sprouts, squash, lotus root, green beans, wax gourd
Mushrooms	flammulina velutipes, laver, oyster mushroom, mushrooms, pleurotus eryngii, kelp
Fruits	orange, watermelon, pineapple, apple, longan, pear, grape, banana, mango, pitaya, peach
Nuts	walnut, peanut, pistachio, sunflower seed, pine nut, chestnut, almond, pumpkin seeds
Beverages	tea, cola, fruit juice, sports drinks

**Table 2:** Characteristics of participants in the study.

Characteristic	Total (*n* = 100)	Vegetarians (*n* = 36)	Omnivores (*n* = 64)	*p*
Age (years), mean ± SD	32.8 ± 4.8	33.6 ± 5.1	32.3 ± 4.6	0.091
Height (cm), mean ± SD	167.0 ± 7.4	165.0 ± 7.3	168.0 ± 7.2	0.086
Weight (kg), mean ± SD	65.1 ± 5.9	59.5 ± 4.9	68.1 ± 6.3	0.051
Body mass index (kg/m2), mean ± SD	23.1 ± 3.1	21.8 ± 2.5	23.8 ± 3.2	0.063
Gender, *n* (%)				
Male	48 (48.0)	16 (44.4)	32 (50.0)	
Female	52 (52.0)	20 (55.6)	32 (50.0)	
Education Level, *n* (%)				
Junior middle school	11 (11.0)	4 (11.1)	7 (10.9)	
Senior high school	29 (29.0)	10 (27.8)	19 (29.7)	
Vocational-technical school	42 (42.0)	16 (44.4)	26 (40.6)	
University degree or above	18 (18.0)	6 (16.7)	12 (18.8)	
Employment status, *n* (%)				
Employed	72 (72.0)	21 (58.3)	51 (79.7)	
Unemployed	28 (28.0)	15 (41.7)	13 (20.3)	

**Table 3:** Reproducibility study: Mean daily food intake, difference and correlation coefficients for the comparison between SQFFQ1 and SQFFQ2.

Food Groups Energy Macronutrients	Vegetarians									Omnivores								
	SQFFQ1			SQFFQ2			D (%)	*p*	*r*	SQFFQ1			SQFFQ2			D (%)	*p*	*r*
	Mean ± SD	Median	IQR	Mean ± SD	Median	IQR				Mean ±SD	Median	IQR	Mean ± SD	Median	IQR			
Rice (g/day)	86.7 ± 14.6	85.6	34.4	89.4 ± 14.4	85.7	42.9	−3.1	0.162	0.68 *	209.3 ± 20.2	200	64.3	199.1 ± 26.6	200	57.15	4.9	0.588	0.64 *
Porridge (g/day)	29.4 ± 3.9	25.7	26.3	28.6 ± 3.1	24.9	20.8	2.7	0.527	0.71 *	40.3 ± 6.2	38.9	26.6	38.1 ± 5.2	37.1	23.1	5.5	0.667	0.55 *
Flour food (g/day)	31.1 ± 6.3	26.7	24.6	32.5 ± 7.2	29.9	22.4	−4.5	0.552	0.53 *	60.6 ± 7.2	58.6	27.95	63.6 ± 5.5	67.1	22.55	5.0	0.173	0.56 *
Buns (g/day)	42.3 ± 8.4	42.9	35.7	43.1 ± 6.4	37.1	27.1	−1.9	0.656	0.55 *	45.2 ± 6.4	42.9	23.1	48.1 ± 5.4	48.6	25.45	−6.4	0.159	0.47 *
Pastry food (g/day)	15.4 ± 4.3	14.3	11.4	14.3 ± 4.8	17.1	10	7.1	0.689	0.64 *	29.2 ± 3.8	27.1	13.6	31.2 ± 2.9	30.4	15	−6.8	0.161	0.67 *
Fried food (g/day)	13.4 ± 5.8	11.3	8.7	14.7 ± 5.3	13.3	9.2	9.7	0.082	0.57 *	22.6 ± 6.1	23.3	13.25	24.1 ± 6.5	23.2	15.05	−6.6	0.071	0.65 *
Coarse cereals (g/day)	226.5 ± 22.7	231.6	56.4	224.9 ± 23.1	235.7	100	0.7	0.894	0.81 *	102.1 ± 12.4	72.9	28.6	100.3 ± 14.8	92.9	62.9	1.8	0.085	0.44 *
Potato (g/day)	83.5 ± 11.9	60	25.7	80.0 ± 10.1	42.9	31.4	5.5	0.168	0.65 *	60.2 ± 11.7	68.6	24.3	60.4 ± 11.5	51.4	30.9	0.3	0.198	0.71 *
Dairy (g/day)	214.3 ± 24.7	228.6	235.2	217.1 ± 21.3	257.1	142.8	1.3	0.792	0.58 *	248.9 ± 32.7	257.1	84.25	250.9 ± 38.0	242.8	49.95	0.8	0.167	0.64 *
Eggs (g/day)	38.7 ± 9.9	28.6	23.5	39.3 ± 10.1	37.6	21.4	−1.5	0.789	0.45 *	55.4 ± 7.9	42.9	25.7	55.0 ± 8.2	51.4	22.8	0.7	0.192	0.63 *
Red meat (g/day)	0.0	0.0	0.0	0.0	0.0	0.0	-	-	-	57.9 ± 10.1	60	25	54.5 ± 11.1	71.4	28.5	−5.9	0.086	0.84 *
Poultry (g/day)	0.0	0.0	0.0	0.0	0.0	0.0	-	-	-	63.9 ± 9.9	49.3	24.8	60.9 ± 10.1	59.7	31.6	4.7	0.076	0.83 *
Processed meat (g/day)	0.0	0.0	0.0	0.0	0.0	0.0	-	-	-	10.7 ± 2.6	17.2	14.25	9.0 ± 2.3	16.7	10.6	−15.8	0.089	0.79 *
Freshwater fish (g/day)	0.0	0.0	0.0	0.0	0.0	0.0	-	-	-	43.3 ± 7.3	45.7	16.5	45.8 ± 7.9	48.6	16.4	−5.8	0.075	0.73 *
Seafood (g/day)	0.0	0.0	0.0	0.0	0.0	0.0	-	-	-	22.5 ± 3.0	23.5	12.3	23.2 ± 4.1	48.7	46.2	−3.1	0.089	0.78 *
Bean products (g/day)	49.8 ± 8.1	52.2	25.6	51.3 ± 7.9	51.4	27.1	3.0	0.064	0.63 *	37.5 ± 6.6	34.3	24.95	39.3 ± 5.6	51.4	23.3	−4.8	0.078	0.54 *
Dark vegetables (g/day)	212.4 ± 22.5	228.6	88.2	214.2 ± 24.9	214.3	42.9	−0.8	0.793	0.85 *	132.2 ± 25.2	125.7	67.1	132.1 ± 25.8	128.6	49.35	0.1	0.852	0.52 *
Light vegetables (g/day)	121.3 ± 14.1	78.8	26.8	123.7 ± 13.9	87.3	28.1	2.9	0.689	0.84 *	78.9 ± 12.2	72.3	30.1	79.2 ± 12.9	73.8	30.35	−0.4	0.796	0.61 *
Mushrooms (g/day)	56.1 ± 9.8	46.3	22.9	55.3 ± 9.8	57.1	22.8	1.4	0.295	0.65 *	27.9 ± 4.4	27.1	16.7	29.4 ± 3.4	31.4	10.5	−5.3	0.079	0.67 *
Fruits (g/day)	285.4 ± 28.5	279.5	88.9	286.3 ± 29.6	278.6	111.4	−0.3	0.916	0.88 *	135.2 ± 23.8	128.6	76.4	132.4 ± 25.7	150	63.55	2.1	0.376	0.47 *
Nuts (g/day)	20.8 ± 3.1	20	12.2	22.4 ± 3.7	21.4	10	−7.7	0.066	0.46 *	26.8 ± 3.4	27.1	12.8	26.2 ± 3.6	25.7	17.9	2.2	0.179	0.53 *
Beverages (ml/day)	35.4 ± 5.1	25.7	21.4	33.3 ± 5.4	35.7	21.5	−5.9	0.054	0.49 *	43.2 ± 12.6	35.7	22.9	44.9 ± 11.1	42.9	28.5	−3.9	0.091	0.71 *
Energy (kcal/day)	2958.8 ± 428.3	2964.3	607.9	3018.9 ± 312.5	3053.6	425.9	−1.9	0.687	0.56 *	2966.3 ± 313.6	2974.5	445.5	3192.9 ± 343.8	3184.9	446.6	−7.6	0.085	0.82 *
Protein (g/day)	122.3 ± 17.8	120.2	22.9	124.6 ± 12.9	124.9	12.7	−1.9	0.072	0.76 *	127.7 ± 12.4	128.5	15.95	146.5 ± 17.5	147.9	24.55	−14.7	0.054	0.71 *
Fat (g/day)	129.1 ± 26.7	134.4	38	132.4 ± 21.5	135.3	30.8	−2.6	0.069	0.68 *	120.0 ± 18.3	119.3	24.6	130.3 ± 19.9	131.9	22.85	−8.5	0.059	0.82 *
Carbohydrates (g/day)	380.2 ± 43.5	376.9	51.5	386.3 ± 52.2	385.7	78.1	−1.6	0.891	0.84 *	379.3 ± 37.9	375.9	51.75	395.6 ± 45.9	401.5	58.4	−4.3	0.856	0.78 *

Note: SQFFQ1 represents the results of the first semi-quantitative food frequency questionnaire; SQFFQ2 represents the results of the second semi-quantitative food frequency questionnaire; IQR represents the interquartile range, IQR = 75th percentile−25th percentile; D represents difference, difference (%) = (SQFFQ1−SQFFQ2)/SQFFQ1*100; *r* represents Pearson correlation coefficients; * represents *p* < 0.05.

**Table 4:** Validity study: mean daily food intake, difference and correlation coefficients for the comparison between SQFFQ1 and 24 HRs.

Food Groups Energy Macronutrients	Vegetarians										Omnivores									
	SQFFQ1			24 HRs			D (%)	*p*	*r*	*r*_adj_	SQFFQ1			24 HRs			D (%)	*p*	*r*	*r*_adj_
	Mean ±SD	Median	IQR	Mean ± SD	Median	IQR					Mean ±SD	Median	IQR	Mean ± SD	Median	IQR				
Rice (g/day)	86.7 ± 14.6	85.6	34.4	95.0 ± 10.7	90.9	35.4	−9.5	0.273	0.63 *	0.61 *	209.3 ± 20.2	200	64.3	195.2 ± 23.5	190	36.65	6.5	0.178	0.63 *	0.62 *
Porridge (g/day)	29.4 ± 3.9	25.7	26.3	29.3 ± 4.6	28.3	22.1	0.3	0.062	0.62 *	0.59 *	40.3 ± 6.2	38.9	26.6	39.6 ± 3.5	37.9	20.65	1.7	0.086	0.54 *	0.52 *
Flour food (g/day)	31.1 ± 6.3	26.7	24.6	33.1 ± 5.5	34.7	29.8	−6.5	0.061	0.54 *	0.51 *	60.6 ± 7.2	58.6	27.95	63.8 ± 11.2	60	19.35	−5.2	0.163	0.55 *	0.51 *
Buns (g/day)	42.3 ± 8.4	42.9	35.7	43.0 ± 16.6	40	25	−1.7	0.085	0.56 *	0.55 *	45.2 ± 6.4	42.9	23.1	47.2 ± 10.9	50	26.7	−4.4	0.081	0.47 *	0.43 *
Pastry food (g/day)	15.4 ± 4.3	14.3	11.4	13.4 ± 5.2	16.7	10	13.3	0.354	0.62 *	0.60 *	29.2 ± 3.8	27.1	13.6	32.1 ± 5.6	30	13.4	−9.9	0.058	0.68 *	0.67 *
Fried food (g/day)	13.4 ± 5.8	11.3	8.7	12.6 ± 3.4	12.3	4.8	13.5	0.354	0.52 *	0.51 *	22.8 ± 6.1	23.3	13.25	22.6 ± 7.5	21	13.4	0.8	0.079	0.63 *	0.61 *
Coarse cereals (g/day)	226.5 ± 22.7	231.6	56.4	217.6 ± 20.8	233.3	133.3	3.9	0.857	0.74 *	0.72 *	102.1 ± 12.4	72.9	28.6	98.2 ± 17.4	93.3	43.35	3.8	0.264	0.46 *	0.46 *
Potato (g/day)	83.5 ± 11.9	60	25.7	62.3 ± 12.8	60	23.3	1.9	0.261	0.63 *	0.59 *	60.2 ± 11.7	68.6	24.3	58.8 ± 13.2	66.7	34.95	2.3	0.169	0.72 *	0.68 *
Dairy (g/day)	214.3 ± 24.7	228.6	235.2	203.6 ± 30.5	216.7	150	5.0	0.393	0.54 *	0.54 *	268.9 ± 32.7	257.1	84.25	248.6 ± 38.4	250	83.3	7.5	0.175	0.86 *	0.85 *
Eggs (g/day)	38.7 ± 9.9	28.6	23.5	40.8 ± 5.3	36.7	33.3	−5.4	0.384	0.46 *	0.44 *	55.4 ± 7.9	42.9	25.7	53.5 ± 22	50	40	3.4	0.094	0.62 *	0.61 *
Red meat (g/day)	0.0	0.0	0.0	0.0	0.0	0.0	-	-	-	-	57.9 ± 10.1	60	25	67.3 ± 24.3	76.7	31.65	−16.4	0.052	0.54 *	0.54 *
Poultry (g/day)	0.0	0.0	0.0	0.0	0.0	0.0	-	-	-	-	63.9 ± 9.9	49.3	24.8	62.4 ± 25.1	62.8	26.25	2.3	0.376	0.52 *	0.49 *
Processed meat (g/day)	0.0	0.0	0.0	0.0	0.0	0.0	-	-	-	-	15.7 ± 2.6	17.2	14.25	17 ± 5.7	17.7	8.5	−17.2	0.051	0.61 *	0.59 *
Freshwater fish (g/day)	0.0	0.0	0.0	0.0	0.0	0.0	-	-	-	-	46.3 ± 7.3	45.7	16.5	43.9 ± 5.3	46.7	20	5.2	0.065	0.68 *	0.65 *
Seafood (g/day)	0.0	0.0	0.0	0.0	0.0	0.0	-	-	-	-	22.5 ± 3.0	23.5	12.3	25.2 ± 3.9	45	53.6	−12	0.057	0.47 *	0.44 *
Bean products (g/day)	49.8 ± 8.1	52.2	25.6	52.9 ± 7.8	53.3	23.4	−6.2	0.164	0.53 *	0.52 *	37.5 ± 6.6	34.3	24.95	41.8 ± 9.6	43.3	20	−11.5	0.058	0.74 *	0.70 *
Dark vegetables (g/day)	212.4 ± 22.5	228.6	88.2	222.0 ± 25.2	233.3	66.7	−4.7	0.696	0.82 *	0.82 *	132.2 ± 25.2	125.7	67.1	120.1 ± 31.3	125.3	50	9.2	0.067	0.58 *	0.58 *
Light vegetables (g/day)	121.3 ± 14.1	78.8	26.8	122.1 ± 15.3	78.6	40	1.5	0.098	0.76 *	0.75 *	78.9 ± 12.2	72.3	30.1	76.5 ± 10.6	76.2	33.5	3.0	0.292	0.51 *	0.48 *
Mushrooms (g/day)	56.1 ± 9.8	46.3	22.9	54.7 ± 5.8	53.3	23.3	2.5	0.075	0.57 *	0.53 *	27.9 ± 4.4	27.1	16.7	24.0 ± 6.1	26.7	18.35	13.9	0.053	0.62 *	0.61 *
Fruits (g/day)	285.4 ± 28.5	279.5	88.9	274.4 ± 22	266.7	100	3.8	0.087	0.68 *	0.65 *	135.2 ± 23.8	128.6	76.4	127.3 ± 28.2	140	76.6	5.8	0.476	0.46 *	0.44 *
Nuts (g/day)	20.8 ± 3.1	20	12.2	20.5 ± 2.8	20	14	1.5	0.694	0.47 *	0.43 *	26.8 ± 3.4	27.1	12.8	30.8 ± 4.1	26.7	13.3	−14.9	0.055	0.43 *	0.40 *
Beverages (ml/day)	35.4 ± 5.1	25.7	21.4	31.6 ± 5.4	30	20	10.7	0.059	0.49 *	0.48 *	43.2 ± 12.6	35.7	22.9	46.5 ± 11.1	43.3	26.7	−7.6	0.053	0.69 *	0.65 *
Energy(kcal/day)	2958.8 ± 428.3	2964.3	607.9	3058.9 ± 286.9	3105	482.5	−3.4	0.578	0.68 *	-	2966.3 ± 313.6	2974.5	445.5	3115.0 ± 273.9	3106.9	463.9	−5.0	0.795	0.71 *	-
Protein(g/day)	122.2 ± 17.8	120.2	22.9	126.2 ± 11.2	129.1	18.9	−3.3	0.269	0.75 *	0.71 *	127.7 ± 12.4	128.5	15.95	143.2 ± 14.6	143.9	21.55	−12.7	0.063	0.68 *	0.66 *
Fat(g/day)	129.1 ± 26.6	134.4	38	135.3 ± 20.0	134	25.7	−4.8	0.466	0.81 *	0.76 *	120.0 ± 18.3	119.3	24.6	128.3 ± 15.1	125.8	24.6	−6.9	0.171	0.78 *	0.74 *
Carbohydrates(g/day)	380.2 ± 43.5	376.9	51.5	388.1 ± 54.3	387.9	80.6	−2.1	0.383	0.83 *	0.82 *	379.3 ± 37.9	375.9	51.75	385.6 ± 35.6	384.1	48.8	−1.6	0.069	0.84 *	0.83 *

Note: SQFFQ1 represents the results of the first semi-quantitative food frequency questionnaire; 24 HRs represents the average of 24 h dietary records for 3 consecutive days; IQR represents interquartile range, IQR = 75th percentile−25th percentile; D represents difference, difference (%) = (SQFFQ1−24 HRs)/SQFFQ1*100; *r* represents Pearson correlation coefficients; *r*_ad__j_ represents energy-adjusted correlation coefficient; * represents *p* < 0.05.
